# Design of a randomised acupuncture trial on functional neck/shoulder stiffness with two placebo controls

**DOI:** 10.1186/1472-6882-14-246

**Published:** 2014-07-16

**Authors:** Nobuari Takakura, Miho Takayama, Akiko Kawase, Ted J Kaptchuk, Jian Kong, Hiroyoshi Yajima

**Affiliations:** 1 Department of Acupuncture and Moxibustion, Faculty of Health Sciences, Tokyo Ariake University of Medical and Health Sciences, 2-9-1 Ariake, Koto-ku, Tokyo 135-0063, Japan; 2 Department of Physiology, Showa University School of Medicine, 1-5-8 Hatanodai, Shinagawa-ku, Tokyo, Japan; 3 Japan School of Acupuncture, Moxibustion, and Physiotherapy, 20-1 Sakuragaokacho, Shibuya-ku, Tokyo, Japan; 4 Program in Placebo Studies & Therapeutic Encounter, Beth Israel Deaconess Medical Center, Harvard Medical School, 330 Brookline Avenue, Boston, MA 02215, USA; 5 Department of Psychiatry, Massachusetts General Hospital, Harvard Medical School, Charlestown, MA 02129, USA

**Keywords:** Acupuncture, Placebo, Neck stiffness, Shoulder stiffness, Double-blind, Randomised, placebo-controlled trial

## Abstract

**Background:**

Functional neck/shoulder stiffness is one of the most well-known indications for acupuncture treatment in Japan. There is little evidence for the effectiveness of acupuncture treatment for functional neck/shoulder stiffness. Research using two different placebos may allow an efficient method to tease apart the components of real acupuncture from various kinds of ‘non-specific’ effects such as ritual with touch or ritual alone. Herein, we describe a protocol of an ongoing, single-centre, randomised, placebo-controlled trial which aims to assess whether, in functional neck/shoulder stiffness, acupuncture treatment with skin piercing has a specific effect over two types of placebo: skin-touching plus ritual or ritual alone.

**Methods:**

Six acupuncturists and 400 patients with functional neck/shoulder stiffness are randomly assigned to four treatment groups: genuine acupuncture penetrating the skin, skin-touch placebo or no-touch placebo needles in a double-blind manner (practitioner-patient blinding) or no-treatment control group. Each acupuncturist applies a needle to each of four acupoints (Bladder10, Small Intestine14, Gallbladder21 and Bladder42) in the neck/shoulder to 50 patients. Before, immediately after and 24 hours after the treatment, patients are asked about the intensity of their neck/shoulder stiffness. After the treatment, practitioners and patients are asked to guess whether the treatment is “penetrating”, “skin-touch” or “no-touch” or to record “cannot identify the treatment”.

**Discussion:**

In addition to intention-to-treat analysis, we will conduct subgroup analysis based on practitioners’ or patients’ guesses to discuss the efficacy and effectiveness of treatments with skin piercing and various placebo controls. The results of practitioner and patient blinding will be discussed. We believe this study will further distinguish the role of different components of acupuncture.

**Trial registration:**

Current Controlled Trial ISRCTN76896018

## Background

Functional neck/shoulder stiffness is the second most frequent subjective symptom next to lumbago [1] and one of the most well-known indications for acupuncture treatment in Japan. Functional neck/shoulder stiffness refers to discomfort, tightness or rigidity in the muscles supporting the neck [2,3], without any specific diseases that cause such complaints. In routine clinical care with acupuncture, patients well experience that needle penetration at acupoints effectively reduces tension and discomfort in the neck and shoulder. The mechanisms underlying the effectiveness of acupuncture for functional neck/shoulder stiffness are unclear. Stiffness and its resultant discomfort in the muscles might be mitigated through neuronal circuits that are similar to those to modulate pain and its resultant unpleasantness [4,5], through the improvement of regional blood circulation [6,7] or through the suppression of moto-neuron activity that innervates the stiff muscles [8]. Some have argued that acupuncture is primarily a placebo effect [9]. There is little evidence for the efficacy of acupuncture treatment for functional neck/shoulder stiffness, and no randomised, placebo-controlled trials (RCTs) investigating acupuncture under double-blind (practitioner-patient) conditions has been reported.

Single-blind RCTs using single-blind acupuncture placebo/sham devices have been considered the most rigorous method for investigating the efficacy of acupuncture [10-13]. However, single-blind design has the potential to produce biases from unmasked practitioners, and evidence obtained under single-blind conditions is arguably insufficiently rigorous [14-21]. Practitioner blinding has been absent in acupuncture research and it may be crucial in acupuncture research to meet the gold standard of double-blind RCTs whereby the specific effects of interventions can be distinguished under double-blinding conditions from non-specific effects [22]. In acupuncture research, however, the gold standard was considered almost impossible to achieve because blinding an acupuncturist is very difficult due to the nature of the procedure [10-15]. To solve this methodological difficulty, we designed two types of non-penetrating placebo needles: one where the tip touches the skin (skin-touch placebo needles), and another where the needle tip does not reach the skin (no-touch placebo needles). We also designed a matched genuine penetrating acupuncture needle. We have evaluated them for use in clinical settings [23-29]. In this study beside genuine penetrating acupuncture we will have two placebo acupuncture controls and an additional no-treatment control. Our two placebos will allow disentanglement to what extent skin-touching plus ritual or ritual alone contributes to the non-specific effects of placebo acupuncture and allow for a precise estimate of the mechanism whereby acupuncture may work in clinical practice.

In this study, we enrol patients who had functional neck/shoulder stiffness not due to any specific disease. To determine the efficacy of acupuncture for neck/shoulder stiffness, we use the three types of aforementioned needles to perform a randomised, double-blind (practitioner-patient), placebo-controlled study. The primary aims of the present study are to assess whether acupuncture treatment with skin piercing is superior to two different placebo treatments one with skin-touching plus ritual and one with ritual alone under double-blind conditions. The two placebo controls will allow for a more precise estimate of the components of acupuncture treatment, what might be a superior placebo. The no-treatment control will allow a determination as to whether any of the treatments are superior to natural progression, spontaneous remission or regression to the mean.

## Methods

This ongoing study started from January 2011 and will complete in March 2015. The study protocol was approved by the Ethics Committee of Tokyo Ariake University of Medical and Health Sciences. The study protocol followed CONSORT recommendations. The trial has been registered with the ISRCTN76896018 [30].

### The acupuncture needles

We use three types of needles sterilized with gaseous ethylene oxide for a double-blind study design: 1) penetrating needles that can pierce the skin; 2) skin-touch placebo needles, the tip of which can touch against the skin but cannot penetrate it; 3) no-touch placebo needles, the tip of which cannot reach the skin. The appearances of these three types of needles are indistinguishable. The diameter of the needles is 0.18 mm. The insertion depth of the penetrating needle is 5 mm. A pedestal is used for the needles. These needles developed for the research employing Japanese style acupuncture have been described in detail elsewhere [23-29,31].

### Study design

This is a randomised, double-blind (practitioner-patient blinding), placebo-controlled study performed in a single centre including four arms: treatment with penetrating needles (penetrating treatment), treatment with skin-touch placebo needles (skin-touch placebo treatment), treatment with no-touch placebo needles (no-touch placebo treatment) and control with no treatment (no-treatment control) [23-29].

### Setting and participants

The study is conducting at Japan School of Acupuncture, Moxibustion and Physiotherapy, and Tokyo Ariake University of Medical and Health Sciences, Tokyo, Japan.

We employ six (three males, three females) experienced and licensed acupuncturists, and recruit 400 volunteers with functional neck/shoulder stiffness who are students, teachers and staff at the Japan School of Acupuncture, Moxibustion and Physiotherapy or Tokyo Ariake University of Medical and Health Sciences. The aim and details of this study are fully explained to each practitioner and patient using a written consent form. All participants sign written consent form before each treatment starts.

### Interview to exclude volunteers

Before patients enter this study, an assistant interviews volunteers using an interview sheet to record basic demographics and to exclude volunteers who do not meet the inclusion criteria of this study. After the interview sheet is filled out, the assistant measures the patient’s blood pressure and then the assistant checks for any omissions. The assistant says the same words in the same tone to every patient and remain blinded from the nature of the needles throughout the experiment.

#### Inclusion criteria

1. Patients are 18 to 60 years old.

2. Patients have functional neck/shoulder stiffness without pain.

3. Patients have experienced receiving acupuncture and have experienced de-qi.

#### Exclusion criteria

1. Patients have a plan within 24 hours to receive acupuncture, massage, medication or any other treatment for neck/shoulder stiffness.

2. Patients have a plan within 24 hours to do self-care for neck/shoulder stiffness, e.g., exercise, stretching and/or supplements.

3. Patients have been diagnosed with any of the following diseases: cervical spondylosis; cervical hernia of the intervertebral disk; cervicobrachial disorder; thoracic outlet syndrome; hepato-cholecystopathy; high blood pressure; cerebrovascular disease; cardiac disease.

4. Patients have any neurological symptoms, such as paralysis or numbness in the neck, shoulder or upper extremities.

5. Patients diagnosed they had diseases to produce neck/shoulder stiffness according to the last annual medical examination.

6. Systolic blood pressure is over 140 mmHg and/or diastolic pressure is over 90 mmHg just before the treatment.

### Randomisation

**Figure 1:**
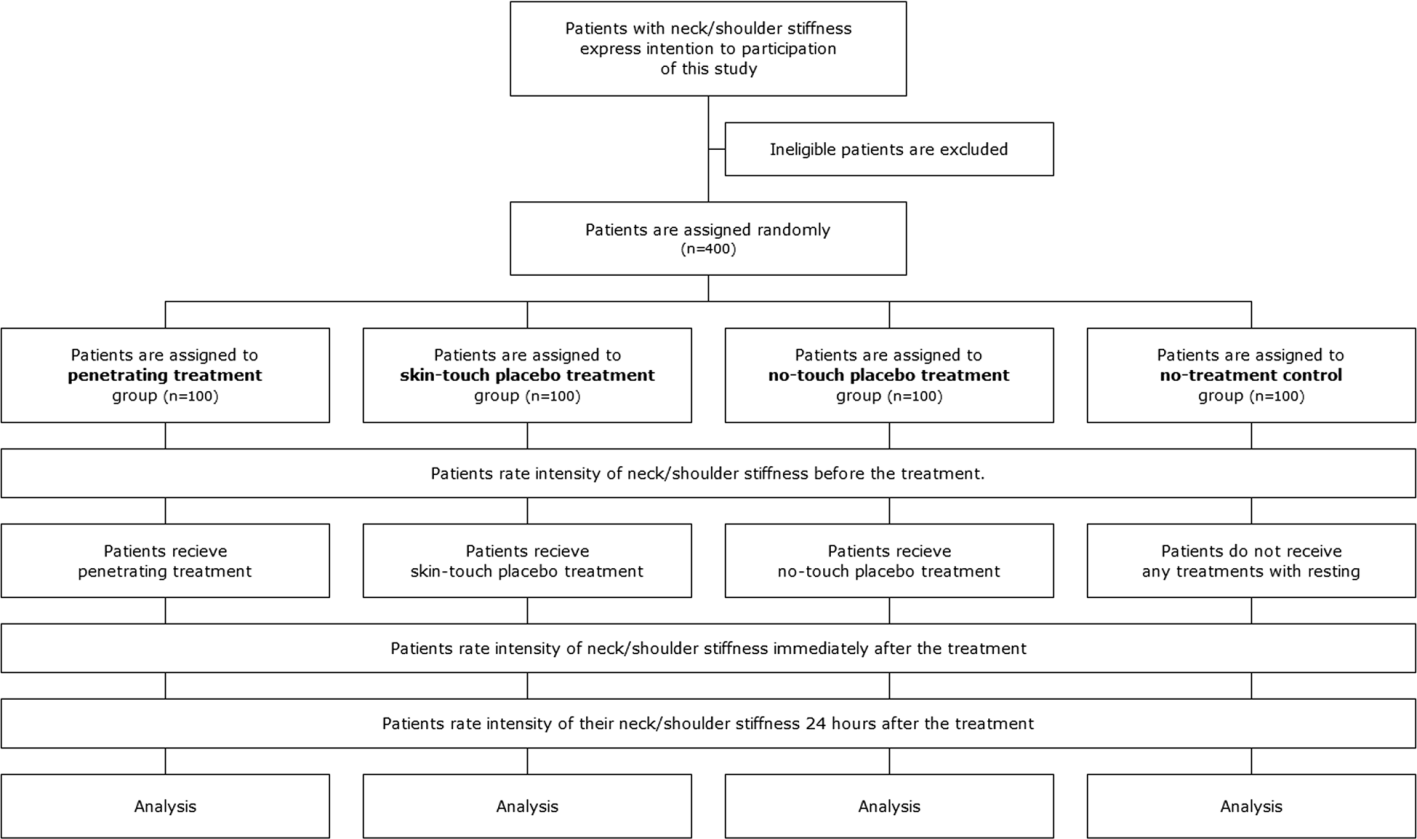
Trial flow diagram.

After screening, qualified patients are randomly assigned to one of the four arms (Figure 1).

Before the trial, we prepared 100 sets of four genuine acupuncture needles for penetrating treatment, 100 sets of four skin-touch placebo needles for skin-touch placebo treatment and 100 sets of four no-touch placebo needles for no-touch placebo treatment. The 400 bags for sterilisation numbered from 1 to 400 were randomly assigned to the penetrating, skin-touch placebo, no-touch placebo, or no-treatment control arm using a table of random numbers that was generated by the RAND function (Microsoft Excel 2003). Four penetrating needles, four skin-touch placebo needles or four no-touch placebo needles were put in a numbered bag corresponding to the assigned treatment type and sealed by the study controller; then they were sterilised with ethylene oxide gas.

The first to the last patient in order are each assigned one of the 400 numbered bags. As a result, 400 patients are randomly assigned to penetrating, skin-touch placebo, no-touch placebo treatment or no-treatment control. Each of the six acupuncturists treats 50 patients consecutively and in order.

### Explanation to patients

At the beginning of the study, each patient is informed the following: “You are going to be randomly assigned to one of the four groups: acupuncture with needles that penetrate the skin, acupuncture with needles that touch the skin but cannot penetrate the skin, acupuncture with needles that cannot reach the skin, or no-treatment control sitting in a massage chair without acupuncture treatment. In case you are assigned to the no-treatment control group, we will treat your stiffness in the neck/shoulder using real needles after completion of the trial if you desire. You have a one-fourth chance of being assigned to the no-treatment control group. The needles are designed to be less painful, so you may not feel pain from treatment”.

### Patients’ evaluation of neck/shoulder stiffness before treatment

Before patients’ evaluation, the assistant makes sure the patients fully understand and that they evaluate the amount of neck/shoulder stiffness only on the right or left side whichever the patients feel is more severe.

The patients record where the strongest stiffness is on the figure in which neck and shoulder is drawn and its duration they have (less than 2 weeks, 2 weeks to 3 months, more than 3 months). The patients rate the intensity of neck/shoulder stiffness by a verbal rating scale (VRS: none, weak, moderate, strong, very strong) and on a 100 mm visual analogue scale (VAS) ranging from 0 (no neck/shoulder stiffness) to 100 (the most severe neck/shoulder stiffness imaginable). The unilateral neck/shoulder in which the patients have stiffness or feel more stiffness than the other side is decided to treat before treatment.

### Needle application and acupoints

Then, the assistant asks the patients to sit on a massage chair, to relax and not to touch or press their own body, especially the neck and shoulder. The patients are asked to indicate the place with the most severe stiffness in the neck/shoulder without touching when the acupuncturist asks them about it.

**Figure 2:**
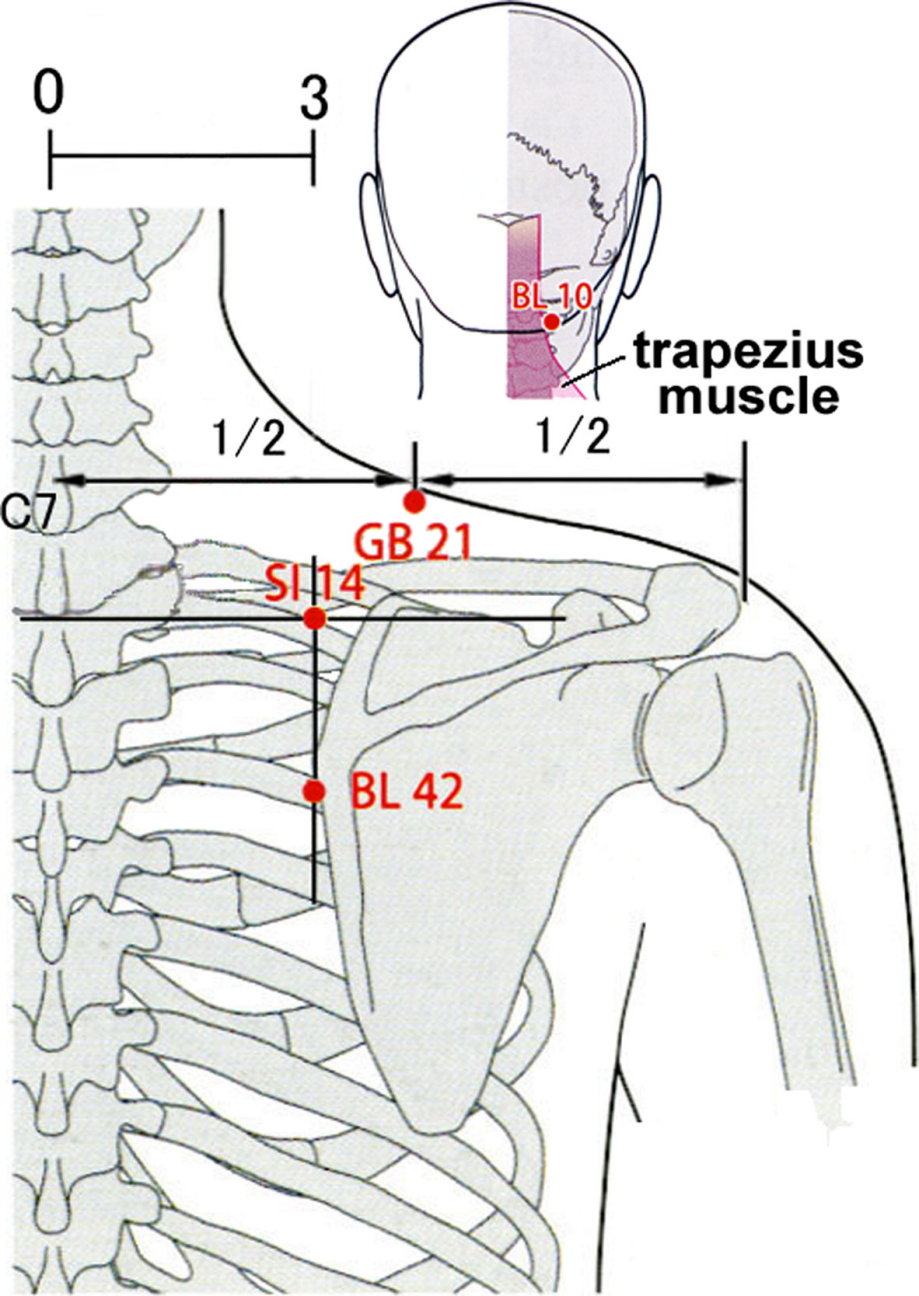
Acupoints for treatment used in this trial.

The assistant tells the acupuncturist that a patient is ready to receive treatment. The acupuncturist enters a treatment booth after an assistant’s signal and she says the following: “Hello, I am going to treat your neck/shoulder stiffness from now on. Please point to where you have the strongest stiffness in the neck or shoulder”. The acupuncturist confirms it and marks the point where the patient indicates. Excluding one of the acupoints BL(Bladder)10, GB(Gallbladder)21, SI(Small Intestine)14 and BL42 in the neck or shoulder (Figure 2) [32], whichever is nearest to the most severe stiffness, the other three acupoints are located and marked to apply needles with a light touch. These acupoints are where many patients with neck/shoulder stiffness complain and commonly used for the treatment of neck/shoulder stiffness.

**Figure 3:**
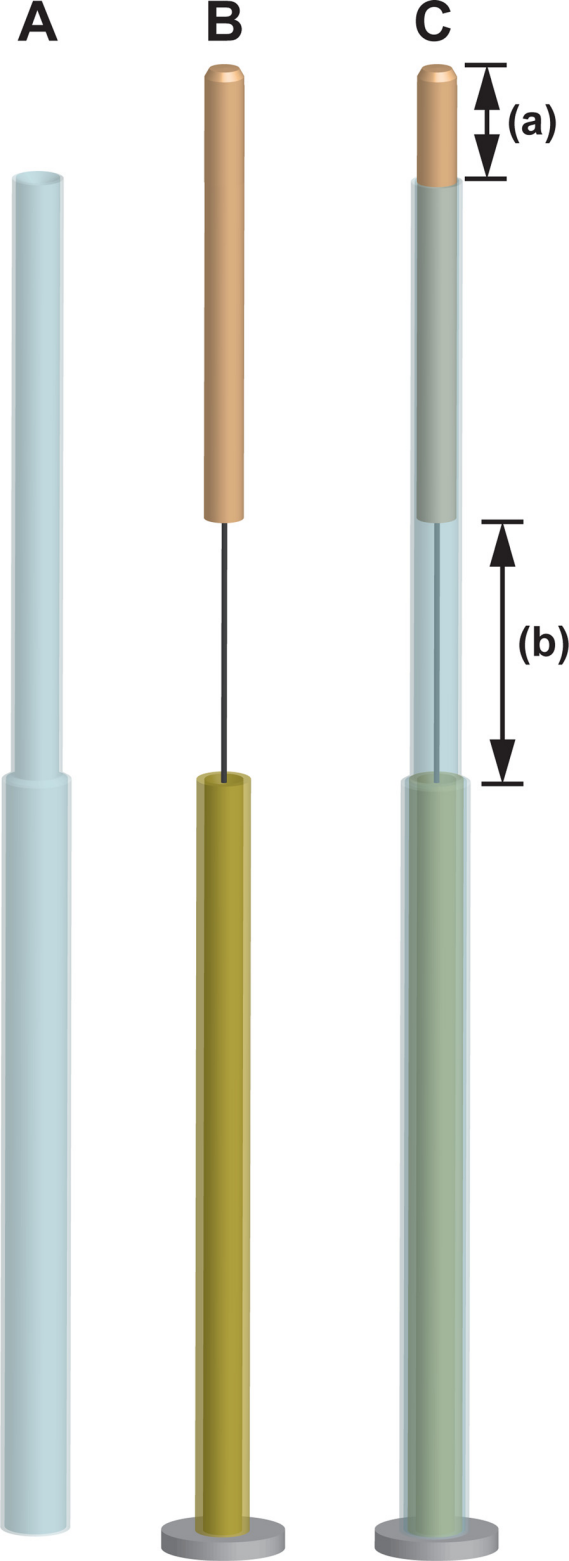
**Double-blind needle assembly and extra guide tube. A**: Extra guide tube. Diameter of upper part of extra guide tube is smaller than that of guide tube needle assembly. **B**: Needle assembly. **C**: Needle assembly covered with extra guide tube. **(a)** indicates insertion depth with tapping-in method. **(b)** indicates whole insertion depth.

The acupuncturist says “I am going to start acupuncture treatment and sterilise the skin with alcohol at and around the acupoints” and sterilises the skin on which the acupoint is located.The assistant prepares a set of four needles, spirits-of-wine cotton and fingerstall, and sets each needle assembly into an extra guide tube (Figure 3). The assistant peels off the seal of the pedestal of each needle and hands the needle to the acupuncturist just before each needle application.

**Figure 4:**
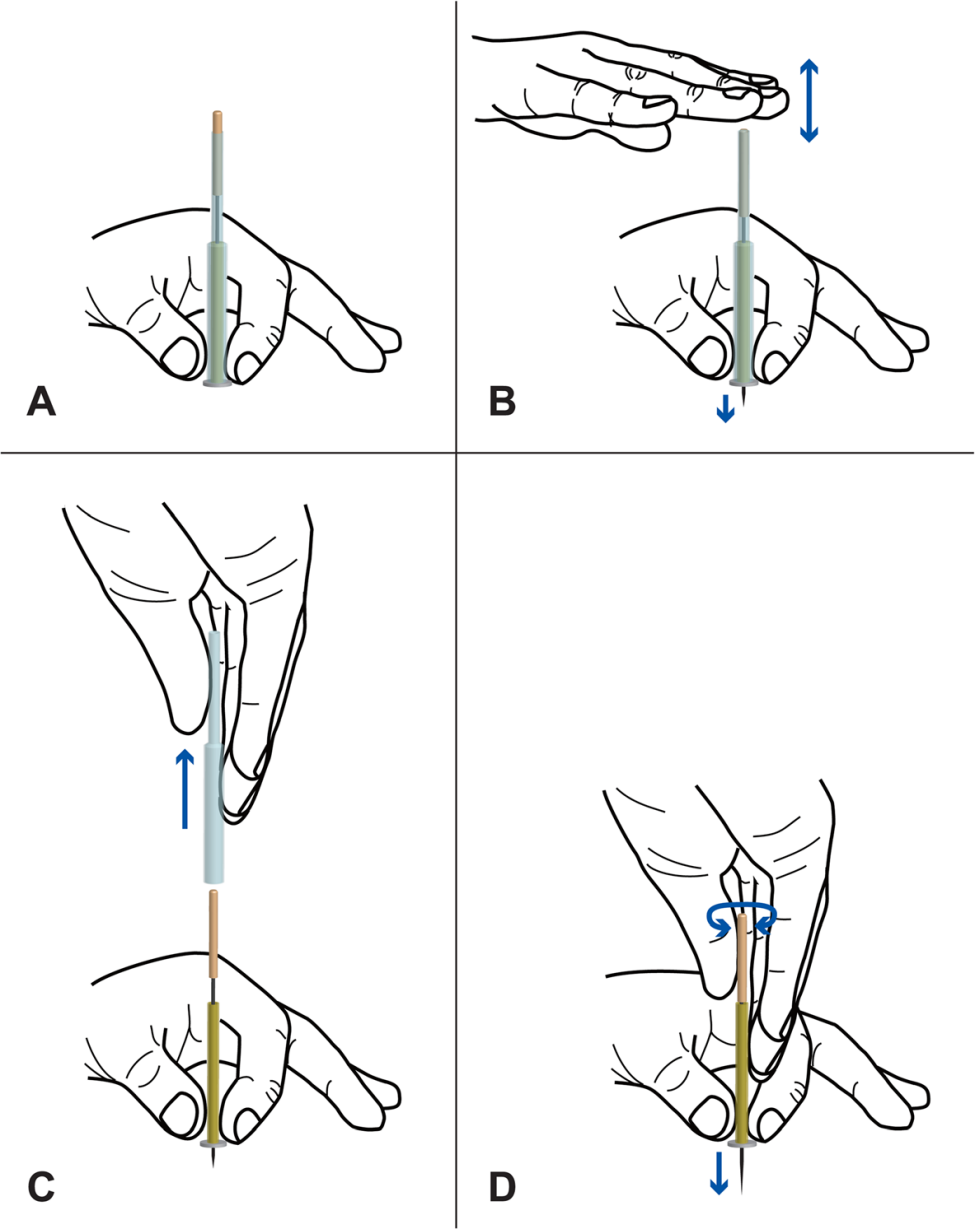
**Course of needle application using extra guide tube in this study. A**: Needle assembly covered with extra guide tube is placed on acupoint. **B**: Upper end of needle handle is tapped (tapping-in method) to penetrate skin. **C**: Extra guide tube is removed from needle assembly after tapping-in method. **D**: Needle is inserted with alternating twirling technique.

Before each needle application, the acupuncturist massages the skin at and around each acupoint with the pulp of index finger with fingerstall (pre-massage). The massage is very light and entails three small strokes. The acupuncturist places a needle on an acupoint, and makes “holding hand” to fix the entire needle assembly with the non-dominant hand (Figure 4A); then with the index finger of the dominant hand, he/she taps the upper end of the needle handle protruding from the top of the extra guide tube to penetrate the skin or the lower stuff in skin-touch and no-touch placebo needles (tapping-in method) (Figure 4B) [29]. After tapping-in method, the acupuncturist removes the extra guide tube (Figure 4C) to insert the needle further by rotating the needle clockwise and counterclockwise alternately (alternating twirling technique) (Figure 4D) [23-28]. Each needle is inserted until the needle tip reaches the target depth, and then the needle is rotated three times.

For each treatment, the acupuncturist applies one needle to BL10, GB21, SI14 and BL42 in that order on the side with the most stiffness. After all four needles are applied, the acupuncturist says to the patient “The needles are going to remain in the body for 10 min (10 min retention)”, and leaves the treatment booth. The assistant says “Please refrain from moving the neck, shoulder or hand. If you feel unbearable pain or something uncomfortable, please let me know. Please relax and do not force yourself to stay awake”.

Ten minutes later, the acupuncturist comes into the treatment booth and says “I am going to remove the needles now”. The acupuncturist returns the needle handle to its initial position by the alternating twirling technique [23-28], removes the entire needle assembly from the skin, and then puts it into an opaque envelope held by the assistant. After removal of each needle, the acupuncturist massages just like pre-massage the place where the needle is applied with three small strokes using the pulp of the index finger with fingerstall to prevent infection with bleeding (post-massage), and then sterilises that area. If there is bleeding, the acupuncturist presses sterilised cotton between the finger and the patient’s skin, and reports the incidence in a questionnaire for practitioners. The needles are removed in the same order in which they are applied.

After treatment is completed, the acupuncturist tells the patient “The acupuncture treatment is done”. Then, the acupuncturist leaves the treatment booth and answers a questionnaire for practitioners.

The assistant observes whether needling have any untoward effects, then asks the patient to dress. In the rare circumstance of slight external bleeding from a point of needle removal the assistant will apply pressure with sterilised cotton to stop bleeding. If there is any internal bleeding after needle removal, the assistant tells the patient that slight internal bleeding occurs and that it will disappear within several days.

### Patients’ evaluation immediately after treatment

The patients rate the intensity of stiffness at the area of strongest stiffness immediately after the treatment by a VRS (none, weak, moderate, strong, very strong) and on a 100 mm VAS ranging from 0 (no neck/shoulder stiffness) to 100 (most severe neck/shoulder stiffness imaginable). Also the patients rate the change in stiffness in the area of strongest stiffness compare with before the treatment by a VRS (clearly worsen, slightly worsen, no changed, slightly improved, clearly improved). When they rate “clearly or slightly worsen”, they rate the change on a 100 mm VAS ranging from −100 (worsened to the most severe stiffness imaginable) to 0 (no change). When they rate “clearly or slightly improved”, they rated the change on a 100 mm VAS ranging from 0 (no change) to 100 (completely cured no stiffness). For the no-treatment control group, after the 10 min of no-treatment period the patients report the same items.

Excluding no-treatment control group, the patients report what they feel during the treatment. If they feel pain from the treatment, they rate pain intensity on a 100 mm VAS ranging from 0 (no pain) to 100 (most severe pain imaginable). If they feel de-qi from the treatment, they rate de-qi intensity on a 100 mm VAS ranging from 0 (no de-qi) to 100 (strongest de-qi imaginable). Further they rate their pleasantness or unpleasantness to the treatment on a 200 mm VAS ranging from −100 (very unpleasant) to 0 (neither pleasant nor unpleasant) to 100 (very pleasant), and rate their satisfaction with the treatment on a 200 mm VAS ranging from −100 (completely unsatisfied) to 0 (neither satisfied nor unsatisfied) to 100 (completely satisfied).

Then, the patients guess whether the treatment is “penetrating”, “skin-touch” or “no-touch” or to record “cannot identify the treatment” and they report their confidence level in their guesses by a VRS (with confidence, with slight confidence, with little confidence, with no confidence) and on a 100 mm VAS ranging from 0 (no confidence) to 100 (full assurance) [24,28,29].

### Patients’ evaluation 24 hours after treatment

The patients rate the intensity of stiffness at the area of strongest stiffness 24 hours after the treatment by a VRS (none, weak, moderate, strong, very strong) and on a 100 mm VAS. When the patients rate at none, they write when it disappears. Also the patients rate the change in stiffness in the area of strongest stiffness compared with before the treatment by a VRS (clearly worsen, slightly worsen, no changed: returned to the pretreatment condition, slightly improved, clearly improved). When their stiffness return to the pretreatment condition, they record when it returns. When they rate “clearly or slightly worsen”, they rate the change on a 100 mm VAS ranging from −100 (the most severe stiffness imaginable) to 0 (no change). When they rate “clearly or slightly improved”, they rate the change on a VAS ranging from 0 (no change) to 100 (no stiffness: completely cured). For no-treatment control group, the patients report the same items.

Excluding no-treatment control group, the patient guess which type of treatments they receive, and they report their confidence in their guess by a VRS (with confidence, with slight confidence, with little confidence, with no confidence) and on a 100 mm VAS ranging from 0 (no confidence) to 100 (full assurance) [24,28,29].

### Practitioners’ guess as to treatment

After completion of treatment with four needles, the acupuncturists guess whether the treatment is “penetrating”, “skin-touch” or “no-touch” or to record “cannot identify the treatment”; they report their confidence in their guesses by a VRS (with confidence, with slight confidence, with little confidence, with no confidence) and on a 100 mm VAS ranging from 0 (no confidence) to 100 (full assurance) [24,27-29]. Additionally, the acupuncturists indicate which clues influence their guesses, such as the patient’s expression, behaviour, external bleeding, internal bleeding, lack of bleeding, reaction to needle insertion, reaction to needle removal and other clues [24,27].

### Outcomes

#### Primary measurements

Improvement of neck/shoulder stiffness score immediately and 24 hours after the treatment are primary outcomes.

#### Secondary outcome measurements

Practitioners’ and patients’ guesses at treatments and their confidence in their guesses and, pain and de-qi felt during treatment by the patients are secondary outcomes.

### Adverse events

The assistant and the acupuncturists monitor the patients for the presence of adverse events, such as pneumothorax, bleeding, haematoma, dizziness, fatigue and needle pain. The assistant also ask the patients to report if they experience any adverse events during and after acupuncture treatment.

### Statistical analysis

Multiple regression analysis will be applied for data analysis. We will use pre- and post-treatment score changes as primary outcome, and treatment modes (penetrating treatment, skin-touch placebo treatment, no-touch placebo treatment and no-treatment control). Additional covariates will include age, gender and other related potential confounders.

In addition, we will define 30% improvement of stiffness intensity on neck/shoulder after treatment as good responders, the chi-square test will be used to determine whether the numbers of improved patients are different in the penetrating, skin-touch placebo and no-touch placebo treatment groups. All statistical analyses will be performed using SPSS (Statistical Package for the Social Sciences, SPSS Inc. Chicago, IL). The 400 bags will be opened by the researchers to reveal and record the nature of the treatments after all 400 treatments have been completed. The assistants who supported the acupuncture treatments will not take part in this work.

## Discussion

Although acupuncture treatment has been administrated for thousands of years, the crucial components of acupuncture remain unclear [33]. This study is a double-blind, randomised, placebo-controlled trial to evaluate the efficacy of acupuncture in treating functional neck/shoulder stiffness. Besides genuine acupuncture (which includes both genuine needle penetration and accompanying ritual of acupuncture), the study utilizes two types of double-blind placebos: skin-touch placebo (which includes the ritual) and no-touch placebo needles (the ritual of acupuncture alone). To clarify the relationship between the cause and the result with minimum bias as possible, we will evaluate the efficacy of acupuncture immediately and 24 hours after treatment. The results will provide high-quality evidence of the efficacy of acupuncture treatment for functional neck/shoulder stiffness with few confounding factors.

Furthermore, we will conduct subgroup analyses on practitioners’ or patients’ guesses in addition to intention-to-treat analysis to understand and discuss the efficacy of needle application, the efficacy of recognition of treatment type, and the relationship between the efficacy of needle application and the efficacy of treatment recognition, by which the efficacy and effectiveness of acupuncture treatment *per se* can be determined. These analyses could provide information on the crucial component of acupuncture such as skin penetration, skin pressure, performance of acupuncture ritual itself as well as the influence of psychological factors between mind of patients and practitioner [33]. Further we will discuss importance of de-qi to produce effect of acupuncture treatment. Thus, the value of acupuncture treatment will be discussed from multiple perspectives and give us new insight that will enable us to implement acupuncture in clinical practice. In addition to improving neck/shoulder stiffness, a critical discussion will be given regarding practitioner and patient blinding and the limitations of the study which is to see short term effect, not a long term follow up.

## Competing interests

NT and the Educational Foundation of Hanada Gakuen possess a U.S. patent 6575992B1, a Canadian patent CA 2339223, a Korean patent 0478177, a Taiwan patent 150135, a Chinese patent ZL00800894.9 (Title: Safe needle, placebo needle and needle set for double blind) and two Japanese patents 4061397 (Title: Placebo needle, and needle set for double-blinding) and 4315353 (Title: Safe needle) on the needles described in this manuscript. NT is a salaried employee of the Educational Foundation of Hanada Gakuen.

## Authors’ contributions

NT, MT, AK and HY have designed the study and written the manuscript. They are also involved in data collection. TK and JK are co-investigators and have contributed to the design and writing of the research protocol. All authors read approved the final manuscript.

## Pre-publication history

The pre-publication history for this paper can be accessed here:

http://www.biomedcentral.com/1472-6882/14/246/prepub
